# Pharmacogenetics or predictive genetics? APOE testing blurs the lines

**DOI:** 10.3389/fphar.2025.1627239

**Published:** 2025-07-21

**Authors:** Stefania Zampatti, Cristina Peconi, Juliette Farro, Fabrizio Piras, Clelia Pellicano, Carlo Caltagirone, Emiliano Giardina

**Affiliations:** ^1^ Genomic Medicine Laboratory UILDM, IRCCS Santa Lucia Foundation, Rome, Italy; ^2^ Neuropsychiatry Laboratory, Department of Clinical Neuroscience and Neurorehabilitation, IRCCS Santa Lucia Foundation, Rome, Italy; ^3^ Department of Clinical and Behavioral Neurology, IRCCS Fondazione Santa Lucia, Rome, Italy; ^4^ Department of Biomedicine and Prevention, Tor Vergata University, Rome, Italy

**Keywords:** pharmacogenetics, predictive test, genetic counselling, alzheimer disease, monoclonal antibodies, APOE, ARIA

## Abstract

The integration of pharmacogenetics into personalized medicine enables the optimization of drug selection and dosage, maximizing therapeutic benefits while minimizing the risk of adverse drug reactions. The association between *APOE* alleles and ARIA, a known adverse reaction in Alzheimer’s disease patients treated with anti-amyloid monoclonal antibodies, has led to the inclusion of *APOE* genotyping among conventional pharmacogenetic tests. Given the dual role of *APOE* alleles, the widespread implementation of this genetic test requires caution and should be accompanied by appropriate genetic counselling. *APOE* genotyping is uniquely positioned at the intersection of pharmacogenetics and germline testing: it provides insight not only into drug safety (specifically the risk of Amyloid-Related Imaging Abnormalities) but also into familial risk for developing Alzheimer’s disease. Carriers of risk alleles, especially homozygotes, face the highest risk and require close monitoring. While *APOE* genotyping can inform treatment decisions, it also raises ethical concerns due to the broader implications of disclosing genetic risk information for neurodegenerative diseases. Identifying a high-risk *APOE* genotype in a patient substantially impacts family members. Therefore, patients considered for treatment with anti-amyloid monoclonal antibodies should receive comprehensive pre- and post-test genetic counseling that goes beyond traditional standards, as currently provided for other peculiar tests. Such counseling ensures that patients are adequately informed about potential outcomes, psychological impacts, and familial implications. It also supports ethical decision-making and facilitates truly informed consent, helping to prevent deterministic or overly simplistic interpretations of genetic risk.

## 1 Introduction

Pharmacogenetics (PGt), the study of how genes affect a person’s response to drugs, is a cornerstone of personalized medicine. Coined in 1959, the term “pharmacogenetics” describes the association between genetic variations and drug response (Vogel, 1959). Actually distinguished in pharmacogenetics (PGt) and pharmacogenomics (PGx), which respectively involve the study of variations in DNA sequence and in DNA/RNA characteristics as they relate to drug response (efficacy, toxicity, adverse drug reactions, etc.) (EMA–European Medicines Agency, 2007). By examining individual genetic variants, PGx aims to tailor medical treatments, optimizing drug selection and dosage to maximize therapeutic benefits while minimizing the risk of adverse drug reactions. The convergence of genetic insights with pharmacological principles holds promise for transforming healthcare by enabling more precise and effective therapeutic interventions (Auwerx et al., 2022; Moyer and Black, 2025).

The Clinical Pharmacogenetics Implementation Consortium (CPIC) reports more than 570 gene-drug interactions, involving 121 genes and 300 drugs (CPIC, 2025). Of these, over 170 drug-gene pairs have prescription guidelines included on FDA labels. Given the widespread use of these drugs and the prevalence of the associated genetic variants, it is estimated that at least one-third of the global population has been exposed to at least one prescription medication with a pharmacogenetic indication (Chanfreau-Coffinier et al., 2019; Auwerx et al., 2022). For these reasons, it has been proposed that pre-emptive pharmacogenetic testing should be implemented to integrate pharmacogenetics in primary care widely (Bryan et al., 2024). While the PGx implementation can significantly advance primary care, these tests must be proposed with adequate pre- and post-test genetic counselling. Recent studies underscore the importance of comprehensive pre-test education and counseling in the context of PGx testing (Allen et al., 2022). Ensuring that patients are well-informed before testing empowers them to make thoughtful, personalized decisions about whether or not to proceed with testing. Additionally, pre-test education enhances their ability to interpret the results accurately and engage in meaningful conversations with their healthcare providers. This collaborative understanding is essential for incorporating PGx insights into medication decisions, ultimately supporting safer, more effective, and individualized treatment strategies (Bagautdinova et al., 2022). While *APOE* genotyping can inform treatment decisions, it raises ethical concerns due to the broader implications of disclosing genetic risk information for neurodegenerative diseases. Identifying a high-risk *APOE* genotype in a patient substantially impacts family members. Therefore, patients considered for treatment with anti-amyloid monoclonal antibodies should receive comprehensive pre- and post-test genetic counseling that goes beyond traditional standards (Bagautdinova et al., 2022), as currently provided for other peculiar tests (Pinto et al., 2016).

## 2 Monoclonal antibodies in Alzheimer’s disease treatment

Alzheimer’s disease (AD) is a progressive neurodegenerative disorder and the most common cause of dementia (Valdez-Gaxiola et al., 2024). Characterized by a gradual decline in cognitive abilities—including memory, language, attention, and executive functioning—the disease ultimately results in a significant loss of independence. The key neuropathological features of AD include the accumulation of extracellular amyloid-beta (Aβ) plaques and intracellular neurofibrillary tangles composed of hyperphosphorylated tau protein in the brain. For many years, treatment strategies primarily aimed at managing symptoms through cholinesterase inhibitors and NMDA antagonists, providing only limited and temporary relief without addressing the underlying disease pathology (Hales, 2025). The amyloid hypothesis proposes that the accumulation of Aβ peptides in the brain activates a cascade of events leading to neuronal dysfunction, formation of neurofibrillary tangles, neuronal loss and ultimately, cognitive decline (Hales, 2025; Greenberg et al., 2025). The recent approval of several monoclonal antibodies (mAbs) targeting amyloid-beta marks a significant shift in the therapeutic landscape, offering a more direct approach to modifying the disease’s progression (Rentz et al., 2024; van Dyck, 2018). Key mAbs that have been approved or are under investigation to treat AD are Aducanumab (Aduhelm^®^), Lecanemab (Leqembi^®^), and Donanemab (Kisunla^®^) (Wu et al., 2023; Kim et al., 2025). While these therapies offer potential benefits, they also present challenges, particularly the risk of Amyloid-Related Imaging Abnormalities (ARIA), which appear as changes on brain MRI scans. Genetic factors, especially the apolipoprotein E (*APOE*) genotype, play a significant role in disease progression, treatment response, and the likelihood of ARIA (Greenberg et al., 2025). ARIA typically presents in two main forms: ARIA-E (edema/effusion) and ARIA-H (hemorrhage/hemosiderin deposition). Usually asymptomatic, both ARIA-E and ARIA-H can be revealed by evaluation of Magnetic Resonance Imaging (MRI) scans (van Dyck, 2018; Hales, 2025; Greenberg et al., 2025; Weidauer and Hattingen, 2025).

The incidence of ARIA varies among the different mAbs approved for AD (Kim et al., 2025; Zimmer et al., 2025; Budd Haeberlein et al., 2022; Filippi et al., 2022; van Dyck et al., 2023; Sims et al., 2023).

Several risk factors can modify the likelihood of developing ARIA during treatment with these mAbs. A consistent finding across clinical trials of anti-amyloid mAbs is the significant influence of the *APOE* genotype on the risk and severity of ARIA (Jessen et al., 2024; Jackson et al., 2024; Cummings, 2023), with a gene-dose effect of the *APOE*ε4 allele (Foley and Wilcock, 2024): homozygotes for *APOE*ε4 (carrying two copies) are at the highest risk, followed by heterozygotes (carrying one copy), while non-carriers have the lowest risk for both ARIA-E and ARIA-H (Hales, 2025; Salloway et al., 2022) (Table 1).

**TABLE 1:** Comparison of safety profile**s** (Filippi et al., 2022; Zimmer et al., 2025).

*mAb*	*ARIA-E Incidence (%)*		*ARIA-H Incidence (%)*	
	*APOE*	*overall*	*APOE*	*overall*
*Aducanumab*	30-43 (ε4 carriers)	26–36	25 (ε4 carriers)	10–16
	0-20 (ε4 non-carriers)		17 (ε4 non-carriers)	
*Lecanemab*	33 (ε4 hom)	13	39 (ε4 hom)	6–17
	11 (ε4 het)		14 (ε4 het)	
	5 (ε4 non-carriers)		12 (ε4 non-carriers)	
*Donanemab*	41-42 (ε4 hom)	24	20 (ε4 hom)	16–27
	23-24 (ε4 het)		12 (ε4 het)	
	16-15 (ε4 non-carriers)		11 (ε4 non-carriers)	

The increased risk of ARIA in *APOE*ε4 carriers is thought to be due to several factors, including a dysfunction of the neurovascular unit and the consequent dysregulation of the blood-brain barrier. Furthermore, *APOE*ε4 promotes brain inflammation, showing a peculiar neuroinflammatory profile through reactive microglia and proinflammatory cytokine expression. Moreover, *APOE*ε4 is a well-known genetic risk factor for Cerebral Amyloid Angiopathy (CAA), and Aβ plaques formation, increasing neuroinflammation levels that lead to disruptions in the blood-brain barrier (Foley and Wilcock, 2024; Greenberg et al., 2020).

The role of the *APOE*ε2 allele in influencing ARIA risk is less consistently reported than *APOE*ε4. Some studies reported that *APOE*ε2 is associated with specific vasculopathic changes that may lead to vessel rupture, while *APOE*ε4 facilitates vascular amyloid deposition (Greenberg et al., 2025; Lozupone et al., 2025). In this scenario, CAA seems more severe in *APOE*ε2 carriers when compared with *APOE*ε3 homozygotes, but this relationship appears inconsistent among different studies (Nelson et al., 2013; Serrano-Pozo et al., 2015).

Other factors that can increase ARIA risk include higher drug dosages, older age, the presence of pre-existing microhemorrhages on baseline MRI, a history of prior strokes, the use of antithrombotic medications, increased amyloid burden, advanced cerebrovascular disease, and a history of transient ischemic attacks or seizures. The strong association between the *APOE*ε4 allele and ARIA suggests a fundamental connection between the genetic predisposition to AD and the brain’s response to amyloid-clearing therapies, possibly related to the extent of vascular amyloid deposition. Moreover, it lays the basis for a new application of the *APOE* genotyping test that can be used as a pharmacogenetic test in people candidates for mAb therapy (Cummings, 2023).

Current guidelines and recommendations for monitoring and managing ARIA in patients receiving Alzheimer’s mAbs treatments emphasize a multi-faceted approach that includes pre-treatment assessment, regular monitoring during treatment, and specific management strategies based on the characteristics of ARIA and the patient’s clinical status. Because ARIA often presents without symptoms, regular MRI monitoring is performed during treatment with mAbs (Hales, 2025; Jessen et al., 2024). Moreover, artificial intelligence tools have been developed to assist clinicians in the identification of ARIA, improving the safety of mAbs therapies (Hales, 2025; Sima et al., 2024). In the pre-treatment assessment of ARIA risks, the characterization of the *APOE* genotype plays a significant role (Hales, 2025; Jessen et al., 2024). FDA-approved labels for aducanumab, lecanemab, and donanemab reported the suggestion to genotype *APOE* before initiating the treatment (FDA–U.S. Food and Drug Administration, 2021; FDA–U.S. Food and Drug Administration, 2023; FDA–U.S. Food and Drug Administration, 2024). While *APOE*ε4 status is a significant risk factor, the recommended monitoring schedules are generally the same for all *APOE* genotypes, although clinicians may exercise more caution with higher-risk individuals.

## 3 APOE is a non-conventional pharmacogenomic test

Personalized medicine uses PGx data to select therapeutic strategies for the right patient at the right time. The reference clinician performs the pre-test counselling for most PGx tests, not necessarily a geneticist, based on generic guidelines (Bagautdinova et al., 2022). The recent diffusion of some peculiar PGx tests, such as the BRCA test for predicting response to PARP inhibitors, highlights counseling responsibilities (provide appropriate information on genetic aspects, familial risks, etc.) (Pinto et al., 2016). Unlike other PGx tests, *APOE* genotyping has peculiar implications and should be performed carefully. Firstly, some *APOE* genotypes are associated with a different risk of disease and an increased frequency of ARIA. Secondarily, *APOE* alleles can be inherited, and the associated risk of AD can be reflected in family members. For these reasons, the *APOE* genetic test should be considered not only a PGx test, but also a germline genetic test: it should be considered the same way as conventional germline genetic tests. Germline genetic testing is widely recognized as distinct from other diagnostic and prognostic procedures due to a unique characteristic: the significance of the information it provides for the individual and their offspring, relatives, and extended family. This distinctive aspect, known as genetic exceptionalism, has led to the development of various protocols to ensure the responsible integration of genetic testing into clinical practice (Lorenzo et al., 2023).

The *APOE* gene on chromosome 19 has three main alleles: ε2, ε3, and ε4. Each individual inherits one *APOE* allele from each parent, resulting in six possible genotype combinations: ε2/ε2, ε2/ε3, ε2/ε4, ε3/ε3, ε3/ε4, and ε4/ε4. These different alleles produce slightly different forms of the ApoE protein, which have varying effects on cholesterol metabolism (Blanchard et al., 2022; Cho et al., 2020) and are implicated in the development and progression of AD (Abondio et al., 2019; Jia et al., 2020; Reiman et al., 2025).

As expected, allele frequency varies among populations. The *APOE*ε2 allele is the least common of the three, found in approximately 5%–10% of the European population (Kolbe et al., 2023), and is associated with a reduced risk of developing AD. It is possible that an individual with the ε2 allele develops AD, in this case the disease tends to manifest later in life (Valdez-Gaxiola et al., 2024; Corder et al., 1994; Serrano-Pozo et al., 2015; Jia et al., 2020). The *APOE*ε3 is the most prevalent, found in around 74%–88% of the European population (Kolbe et al., 2023) and is generally considered to have a neutral effect on the risk of developing AD (Valdez-Gaxiola et al., 2024; Jia et al., 2020). The *APOE*ε4 allele is more common than *APO*ε2, present in approximately 6%–22% of the European population (Kolbe et al., 2023). It is recognized as the strongest genetic risk factor for late-onset AD, significantly increasing the likelihood of developing the disease and often associated with an earlier age of onset (Jia et al., 2020). The lifetime risk (up to 85 years) for mild cognitive impairment (MCI) or AD has been estimated as 30%–55% for *APOE*ε4 homozygotes, 20%–25% for heterozygous *APOE*ε3/*APOE*ε4, and 10%–15% for *APOE*ε3 homozygotes (Jackson et al., 2024). Interestingly, the risk associated with the *APOE*ε4 allele is age-dependent: AD risk increases progressively with age and subsequently declines after 70–79 years in individuals with a family history of AD, and after 65–74 years in those without a history (Jia et al., 2020). *APOE*ε4 is also associated with a more significant accumulation of amyloid-beta deposits in the brain than non-carriers (Jackson et al., 2024). Furthermore, it has been suggested that the ApoE4 protein might have toxic effects beyond its role in amyloid processing, such as an increased response to stress or injury in the brain (Mahley and Huang, 2012). Understanding that the *APOE* genotype is a risk factor, not a deterministic gene for AD is crucial. Carrying the *APOE*ε4 allele increases susceptibility but does not guarantee that an individual will develop AD. Conversely, individuals without the *APOE*ε4 allele can still develop the disease (Valdez-Gaxiola et al., 2024; Cho et al., 2020). In this context, the *APOE* test can be utilized on diverse subjects, uncovering various implications: in patients with a family history of AD, the *APOE* test (along with an analysis of other AD genes such as *PSEN1*, *PSEN2*, and *APP*) can help estimate the recurrence risk for AD; in AD patients, candidates for mAbs therapy can predict adverse events (ARIA); in healthy individuals, regardless of a family history of AD, it can indicate their susceptibility to AD. Otherwise, performing the *APOE* test in AD patients will provide genetic information about first-degree family members who could be obligate carriers of certain APOE alleles.

## 4 Genetic counselling

As anticipated, defining the *APOE* genotype has consequences that involve the entire family, not limited to the patient. While almost all PGx tests are performed to determine the dosage or the risk of side effects of treatments, *APOE* genotyping can also reveal an individual’s heritable AD risk, besides the risk of ARIA when treated with mAbs. Therefore, *APOE* genetic test should be considered not only a PGx test but also a germline genetic test: the significance of the information it provides for the individual and their offspring, relatives, and extended family (genetic exceptionalism). In this scenario, administering *APOE* genotyping for therapeutic purposes reveals genetic risks for AD that affect the whole family of the AD patient. Given the well-established association between the *APOE*ε4 allele and an increased risk for AD, it is understandable that some laboratories and healthcare providers express caution regarding the ordering or offering of *APOE* testing (i.e., in dyslipidemia genetic panels) (Ison et al., 2024). For over 2 decades, clinicians have raised ethical and practical concerns about disclosing such information to patients, particularly considering its limited clinical utility and the potential for psychological distress (Bird, 1995). Indeed, some individuals have described receiving these results as distressing or even traumatic, particularly in the absence of appropriate genetic counseling (Zallen, 2018). Nevertheless, some studies report that most patients can tolerate this information and derive meaningful benefits, such as guiding decisions related to health behavior modifications and financial planning. The Risk Evaluation and Education for Alzheimer’s Disease (REVEAL) studies, which examined psychological responses to *APOE*ε4 genetic test, found that participants experienced minimal long-term psychological harm (Cassidy et al., 2008; Green et al., 2009). In one arm of the study, the disclosure of *APOE*ε4 status as a risk factor for coronary artery disease was also well tolerated (Christensen et al., 2016). Moreover, evidence suggests that learning one’s genotype has motivated some individuals to adopt healthier lifestyles, including improvements in diet and physical activity (Zallen, 2018).

In all cases where *APOE*ε4 status will be disclosed, pre-test genetic counseling is critical, regardless of context (Green et al., 2009). Clinicians and genetic counsellors should engage in thorough discussions with patients, addressing the clinical and psychosocial implications of testing, clarifying motivations for pursuing genetic information (i.e., antiMAbs administration), and ensuring informed consent that encompasses both the potential benefits and limitations of knowing one’s *APOE* status. An adequate pre-test genetic counselling reduces the risk of anxiety and depression, helping patients deal with possible results, risks associated with one’s genotype, and its heritability. This approach deals with the psychological impact of learning one’s *APOE* status, improving individual knowledge about it and promoting healthier lifestyles. In this context, comprehensive pre-test genetic counselling is essential to equip individuals and caregivers with the necessary information to make informed decisions about *APOE* testing and to prepare them for the potential implications of the results. The information provided should be tailored to the individual’s specific situation, motivations for considering testing, and level of understanding. Furthermore, genetic counselors should discuss the ARIA risks associated with *APOE*ε4 status to provide useful information and possible treatment strategies.

In AD patients, the decision to undergo *APOE* genetic testing involves several critical ethical considerations, each requiring careful attention during the pre-test counselling process. One of the first areas to discuss in genetic counselling is the potential implications for the patient. The genetic counselor ensures that the individual fully understands the purpose of the test, which in this context includes assessing the risk for AD and the potential risk for ARIA if considering treatment with anti-amyloid mAbs (Filippi et al., 2022). The counselling should clearly explain the implications of potential test results, such as an increased or decreased heritable risk for AD and how the *APOE* genotype might influence eligibility for or management during treatment with these emerging therapies. It is also crucial to emphasize the limitations of *APOE* testing, particularly its non-diagnostic nature for AD (Mayeux et al., 1998). Individuals must be informed that testing is entirely voluntary, and they have the right to refuse the test. The discussion should also cover the potential benefits of testing, including informing lifestyle choices, facilitating future planning, determining eligibility for clinical trials, and providing a clearer understanding of ARIA risk associated with specific treatments (in Table 2, propositus-specific considerations are listed). Conversely, the potential risks, such as psychological distress and the possibility of genetic discrimination, should also be thoroughly discussed (Bird, 1995; Zallen, 2018). Providing this information in a culturally and linguistically appropriate format is essential to ensure proper understanding and autonomous decision-making (Uhlmann and Roberts, 2018). The complexity surrounding the dual purpose of *APOE* testing in this context—risk assessment for both the disease and a treatment side effect—adds a significant layer to the informed consent process, requiring comprehensive and tailored explanations. Due to the nature of the AD, it may be necessary that the caregiver and/or family members assist the patient in genetic counselling. In fact, pre-test counselling should also include a discussion about the potential implications of an individual’s *APOE* results for family members. Explaining the inheritance patterns of *APOE* alleles can help family members understand their own potential risk. In this scenario, it is essential to consistently emphasize the probabilistic nature of genetic risk, avoiding deterministic language that might wrongly suggest a definitive outcome. In this context, key issues of genetic counseling have to be adapted to these new applications of *APOE* testing (Goldman et al., 2011; Goldman, 2012; Uhlmann and Roberts, 2018) (Table 2).

**TABLE 2:** Key issues for genetic counseling (Goldman et al., 2011; Goldman, 2012; Uhlmann and Roberts, 2018).

Counselling moment	Options to consider	Suggested conduction	Tips
Patient’s anamnesis	Symptomatic patients	Evaluate the patient’s ability to understand	AD patients can exhibit cognitive impairment. In these cases, deficits in memory and executive functioning (among other cognitive domains) may hinder decisional abilities. Furthermore, cognitive challenges in processing and recalling information can compromise their ability to fully understand the test decision Suggestion: Genetic counseling for symptomatic patients should be performed in the presence of the individual’s caregiver and/or family member. Adopting ad hoc communication strategies can enhance the communication process (i.e., simplifying/reducing amount and types of information provided, using visual aids to help convey probabilities)
	Asymptomatic subjects	Ad hoc preliminary pre-symptomatic protocol	When an asymptomatic subject requires the genetic test, it should be applied according to an ad hoc preliminary protocol based on the International Huntington Association and World Federation of Neurology Research Group on Huntington’s Chorea Guidelines (Hercher et al., 2016) Genetic predictive testing should not be conducted during stressful life events, such aspregnancy. It is essential to ensure that patients have taken adequate time to reflect on their decision and can psychologically cope with receiving the test results
	Prenatal and pediatric testing	Prenatal and pediatric testing for AD should not be performed	Genetic testing for adult-onset conditions should be deferred because it denies the child or future child “the opportunity to make this decision for him/herself as an adult” (International Huntington Association and the World Federation of Neurology Research Group on Huntington's Chorea, 1994)
Patient’s family history	Pedigree drawing	Detailed family history, covering at least three generations	It should include the age of onset of any neurological or psychiatric symptoms, the specific type of dementia, the diagnostic method used, current ages or ages at death—especially of unaffected relatives—and causes of death When possible, medical records should be reviewed to confirm diagnoses of AD. Including information about extended family members may also be helpful, especially in small families or those with a high incidence of early mortality that could obscure a familial pattern of dementia
	Inheritance evaluation	Assess the pedigree for AD risk, AD type, and candidates for testing	The pedigree should be evaluated to assess the AD risk, considering the consistency of family history with EOAD, LOAD, or Mendelian inheritance. Based on this information, the opportunity to perform genetic tests other than APOE should be evaluated. (Goldman et al., 2011)
Evaluation of decisional capacity	Symptomatic and asymptomatic patients	Interdisciplinary evaluation of decisional capacity, considering cognitive function and emotional state	A patient’s decisional capacity can be influenced by neurological conditions, which may variably affect the cognitive and emotional functions necessary for informed decision-making. In neurological disorders such as dementia, progressive cognitive decline often impacts multiple domains critical to decisional capacity, including memory, attention, executive functioning, and the ability to process complex information. These impairments can undermine a patient’s ability to understand relevant facts, appreciate the personal relevance of medical information, and reason through the potential outcomes of different choices. Moreover, even in so-called asymptomatic subjects, signs and symptoms of neuropsychiatric conditions can emerge. Anxiety and depression are common disorders amongfamily members of AD patients, these conditions should be assessed for their potential to compromise decisional capacity
Informed consent	Provide up-to-date informations	Geneticists should be constantly updated to provide up-to-date and useful information to patients	Obtaining truly informed consent for genetic testing presents a range of challenges, particularly due to the evolving nature of genetic knowledge and the complexities associated with genetic conditions. Specifically, the risks, benefits, and limitations of a genetic test may not be fully understood. The clinical utility, predictive value, and potential implications of genetic tests can vary based on advancedemnts in knowledge
	Individual’s feeling	Individuals interpret and assign value to information and uncertainties through the lens of personal, familial, cultural, religious, and spiritual beliefs	The decision to undergo genetic testing is highly individualized, and patients may weigh similar risks and benefits in markedly different ways. Furthermore, various factors, such as neurological or psychiatric conditions, can complicate the process by impairing a patient’s ability to decide, thereby influencing their capacity to fully engage with and understand the information necessary to make an informed choice
	Personal implications	Understanding medical outcomes and psychosocial impacts through an accessible communication process	It is essential that patients not only understand the potential medical outcomes of testing but also consider the broader psychosocial ramifications, including the emotional impact, family dynamics, and potential implications for life, disability, or long-term healthcare. Comprehensive pre-test genetic counseling plays a pivotal role in this process. Genetic counselling is a non-directive communication process that supports patients’ choices. Geneticists should communicate complex genetic information in an accessible manner, assess decisional capacity, and assist patients in navigating the ethical, emotional, and practical dimensions of testing. Through this guidance, patients are better equipped to make informed, values-aligned decisions regarding whether to pursue genetic testing

## 5 Conclusion

The emergence of mAbs targeting amyloid-beta represents a significant advancement in potential AD treatment. Aducanumab, Lecanemab, and Donanemab all function by reducing amyloid plaque burden in the brain, albeit through slightly different mechanisms. Current guidelines for managing patients on these therapies emphasize the importance of pre-treatment assessment, including confirmation of amyloid pathology and *APOE* genotyping, as well as baseline MRI to identify pre-existing conditions. The *APOE* genotype plays a dual role in AD: risk factor and PGx test. In this scenario, the *APOE* genetic test should not be considered only as a PGx test, because it has the potential to harm patients’ health and their whole family. The administration of *APOE* genetic test should be performed in a dedicated genetic counselling process, as appropriate for neurological late-onset genetic disorders. The growing need to perform *APOE* analyses on many patients (ideally all patients who are candidates for mAbs) should encourage the establishment of genetic counseling centers specifically trained for these conditions.

The increasing diffusion of telemedicine can support genetic counselling improvements, making the evaluation of several patients who cannot reach the medical center available. Telemedicine allows healthcare professionals to conduct real-time virtual consultations, perform clinical assessments, monitor patient health metrics remotely, and provide individualized treatment recommendations without requiring the patient to be physically present in a healthcare facility (Weiskirchen S. and Weiskirchen R., 2025; Griffith et al., 2024).

The update of clinical protocols and the development of optimized network of medical centers in the territory will improve the diagnosis and the management of AD patients (Marra et al., 2025). In this context, the application of telemedicine to genetic counselling for *APOE* will promote an adequate administration, ensuring that the ethical standards required for this complex (pharmacogenetic with several secondary implications) genetic test are met. As healthcare systems increasingly adopt telemedicine as a complement to traditional in-person care, its role in promoting health equity, optimizing resource utilization, and improving overall patient outcomes continues to grow.
